# Quantitative comparison of the efficacies and safety profiles of three first-line non-platinum chemotherapy regimens for advanced non-small cell lung cancer

**DOI:** 10.3389/fphar.2022.806728

**Published:** 2022-08-29

**Authors:** Qian-Yu Yang, Lin Zhu, Hong-Xia Liu, Qing-Shan Zheng, Lu-Jin Li

**Affiliations:** Center for Drug Clinical Research, Shanghai University of Traditional Chinese Medicine, Shanghai, China

**Keywords:** NSCLC, non-platinum chemotherapy, OS, ORR, safety, influencing factors

## Abstract

**Objectives:** The purpose of this study was to quantify the efficacies and safety profiles of the three first-line non-platinum chemotherapy regimens recommended in the National Comprehensive Cancer Network guidelines.

**Materials and Methods:** The PubMed and Cochrane Library databases were searched comprehensively, and clinical trials involving patients with advanced non-small cell lung cancer treated with one of three first-line non-platinum regimens (gemcitabine combined with vinorelbine, gemcitabine combined with docetaxel, or gemcitabine alone) were included in the analysis. A parametric proportional hazard survival model was established to analyze the time course of overall survival (OS). The objective response rate (ORR) and incidence rates of grade 3–4 adverse events (AEs) were summarized using a single-arm meta-analysis with a random-effects model.

**Results:** Seventeen studies met the inclusion criteria. Age and performance status (PS) scores were significant predictors of OS. For each 10-years increase in age, mortality risk increased by 18.5%, and the mortality risk increased by 4% for every 10% increase in the proportion of patients with a PS score of 2. After correcting for the above factors, we found that the three first-line non-platinum chemotherapy regimens did not differ based on OS or toxicity.

**Conclusion:** There was no significant difference in OS or toxicity among the three first-line non-platinum chemotherapy regimens. Age and PS scores were significant predictors of OS, and their heterogeneity across different studies should be considered in cross-study comparisons and sample size estimations when designing clinical trials.

## Introduction

Lung cancer is the most common form of cancer and the leading cause of cancer-related mortality worldwide (Bray et al., 2018). Non-small cell lung cancer (NSCLC) accounts for 80–85% of all lung cancer cases (Crinò et al., 2010), and approximately two-thirds of NSCLC cases are advanced or metastatic at the time of diagnosis (Govindan et al., 2006). In recent years, the treatment of advanced NSCLC has progressed, with breakthroughs in pathogenic gene research. Targeted therapy and immunotherapy are the most advanced treatments for NSCLC. However, approximately 50% of patients still choose cytotoxic chemotherapy, which remains an important part of systematic treatment (Arbour and Riely, 2019).

The standard first-line chemotherapy regimen for patients with advanced NSCLC is a combination of platinum and third-generation chemotherapeutic drugs. However, the efficacy of platinum-based chemotherapy regimens in prolonging survival and relieving symptoms is limited, and the use of such regimens has been associated with various adverse reactions (Goffin et al., 2010). In addition, more than half of patients with lung cancer are ≥60 years old at the time of diagnosis (Fry et al., 1996). Older patients tend to have multiple medical comorbidities. These patients often have a poor performance status (PS) and have difficulty tolerating the toxicity of platinum-based chemotherapy. Therefore, a chemotherapy regimen based around third-generation non-platinum drugs with low toxicity is preferable.

Prior studies have most often focused on comparing overall survival (OS), objective response rate (ORR), and toxicity between non-platinum chemotherapy regimens and platinum-containing chemotherapy regimens (Georgoulias et al., 2001a; Gridelli et al., 2003; Laack et al., 2004; Georgoulias et al., 2005; Tan et al., 2005; Esteban et al., 2006; Yamamoto et al., 2006; Rubio et al., 2009). OS and 1-year survival rates of non-platinum chemotherapy regimens are comparable to those of platinum-containing chemotherapy regimens. In contrast, the ORR and toxicity of non-platinum chemotherapy regimens are lower than those of platinum-containing chemotherapy regimens (Georgoulias et al., 2001b; Daddario et al., 2005; Yu et al., 2012; Jiang et al., 2013). Studies comparing non-platinum chemotherapy regimens and platinum-containing chemotherapy regimens are abundant. However, few head-to-head trials and meta-analyses have compared treatment efficacy and toxicity between different third-generation non-platinum chemotherapy regimens. In addition, the National Comprehensive Cancer Network (NCCN) guidelines do not specify which non-platinum chemotherapy regimen should be used under certain circumstances based on expected efficacy rates and toxicity.

In this study, a model-based meta-analysis (MBMA) was performed to compare the efficacy and safety profiles of three first-line non-platinum chemotherapy regimens in patients with advanced NSCLC. MBMA is a new meta-analysis method that combines mathematical modeling and meta-analysis. For example, by establishing a parametric proportional hazard survival model, we can predict OS at 6 months, 1 year, 2 years, and any arbitrary time points not limited to median survival time. Moreover, through the establishment of a covariable model, various influencing factors can be quantitatively analyzed simultaneously, and the effect of these factors on OS can be assessed. This study is expected to provide quantitative information for the clinical treatment of patients with advanced NSCLC.

## Materials and methods

### Search strategy

The PubMed and Cochrane Library databases were searched using a time limit of up to 3 July 2019. Only clinical trials were considered for inclusion, and we only considered studies published in English. Search terms included those related to the target condition (NSCLC) and drug names (gemcitabine, vinorelbine, and docetaxel). For more details on the search strategies, see Supplementary Information.

The inclusion criteria were: 1) clinical trials; 2) patients with advanced or metastatic NSCLC (stage IIIB or stage IV); 3) treated with first-line chemotherapy; 4) non-eligibility for surgery or radiotherapy; and 5) WHO ECOG PS score of 0–2.

The exclusion criteria were: 1) treatment regimens including surgery, radiotherapy, targeted therapy, immunotherapy, or traditional Chinese medicine treatment and 2) no efficacy or safety results reported.

### Data extraction and quality assessment

Excel (Microsoft Office 2016) was used to collect information on study characteristics (author, year of publication, and clinical trial registration number), trial design (drug name, dose, days of administration, planned administration cycles, route of administration, sample size, and blind method), patient characteristics (age, male ratio, PS score, TNM stage, and tumor pathological classification), and clinical outcomes i.e., OS, ORR, and the incidence of adverse events (AEs). The data provided in the graphics were extracted using the GetData Graph Digitizer (version 2.26.0.20). The above information was independently extracted by two researchers and checked by a third researcher. When extracting data from graphics, the error should not exceed 2%. If it exceeds 2%, data extraction needs to be repeated.

The modified Jadad scale was used to evaluate the quality of the eligible studies. A score of 0–3 denoted low quality, and a score of four to eight denoted medium to high quality. Two researchers performed independent evaluations, and a third researcher resolved any disagreements.

### Model development and evaluation

Three parametric survival models with different hazard functions were used to fit the OS data for each treatment arm (Eqs. 1–3) (Holford and Lavielle, 2011). The risk of death increases with time and disease progression (Eq. 4) (Holford and Lavielle, 2011). Eq. 5 represents the conversion between cumulative death risk and survival rate (Holford and Lavielle, 2011). During the model estimation, it is necessary to consider the inter-study variability of β_0_ (Eq. 6), and residual error (Eq. 7) (Owen and Fiedler-Kelly, 2014).Constant

$$\text{h}\left(\text{t}\right)={\text{β}}_{0}$$ (1)

Gompertz

$$\text{h}\left(\text{t}\right)={\text{β}}_{0}\times {\text{e}}^{{\text{β}}_{1}\times \text{t}}$$ (2)

Weibull

$$\text{h}\left(\text{t}\right)={\text{β}}_{0}\times {\text{e}}^{{\text{β}}_{1}\times \text{Ln}\left(\text{t}\right)}$$ (3)

Cumulative hazard function

$$\text{Λ}\left({\text{t}}_{0},\text{ t}\right)=\overset{0}{\underset{\text{t}}{\int }}\text{h}\left(\text{t}\right)$$ (4)

Survival function

$$\text{S}\left(\text{t}\right)={\text{e}}^{-\text{Λ}\left(0,\text{ t}\right)}$$ (5)

Inter-study variability

$${\text{β}}_{\text{i}}={\text{β}}_{\text{typical}}\times {\text{e}}^{{\text{η}}_{\text{i}}}$$ (6)

Residual error

$${\text{Obs}}_{\text{i},\text{j}}={\text{Pred}}_{\text{i},\text{j}}+{\text{SE}}_{\text{i},\text{j}}\times {\text{ε}}_{\text{i},\text{j}}$$ (7)

In Eqs. 1–3, h(t) is the death risk at time t and β_0_ is the initial death risk at the beginning of the trial. In (Eqs. 4, 5), Λ(t_0_, t) is the cumulative death risk from time 0 to t, and S(t) is the survival rate at time t. In (Eq. 6), β_i_ is the initial death risk of an individual study i, β_typical_ is the typical value of the initial overall death risk of the studies, and η_i_ is the inter-study variability, which is assumed to be normally distributed with a mean of 0 and variance of ω^2^. In Eq. 7, Obs_i,j_ is the observed probability of OS at time j in study i, Pred_j,i_ is the corresponding predicted value, and ɛ_j,i_ is the residual at time j in study i, which was weighed using the standard error of the corresponding probability of OS.

After establishing the base model, it is necessary to investigate factors that may have a potential impact on the risk of death, such as age, sex, PS score, TNM stage, and tumor pathological classification. All factors except age were recorded as percentages. Therefore, all five factors were considered as continuous covariates. Eq. 8 shows the method for introducing continuous covariates (Owen and Fiedler-Kelly, 2014).

$${\text{β}}_{\text{i}}={\text{β}}_{\text{typical}}\times {\text{e}}^{\left(\left({\text{COV}}_{\text{i}}-{\text{COV}}_{\text{median}}\right)\times {\text{θ}}_{\text{COV}}+{\text{η}}_{\text{i}}\right)}$$ (8)

Eq. 8 is transformed by introducing covariates into Eq. 6. In Eq. 8, COV_i_ is the value of the covariate of study i, COV_median_ is the median of the covariates of the overall studies, and θ_COV_ is the correction coefficient of the covariates for the initial death risk.

Each covariate was screened in a stepwise manner based on the differences in objective functional value (OFV) between the hierarchical models. Differences in the OFV of 3.84 (χ^2^, *α* = 0.05, df = 1) and 6.63 (χ^2^, *α* = 0.01, df = 1) during the forward inclusion and backward deletion steps were considered statistically significant (Supplementary Tables S4 and S5).

The fitness of the final model was evaluated based on several aspects, including the plausibility and relative standard errors (RSEs) of the parameter estimates, changes in OFV, goodness-of-fit plots, and visual predictive check plots. Leave-one-out cross-validation was used to evaluate the sensitivity of the final model. One set of data was dropped from the entire dataset at a time, and the remaining data were used to fit the final model. Parameter estimates obtained from the datasets were compared to investigate the robustness of the final model.

### Subgroup analysis of overall survival

After the model was constructed, the values of the model parameters and their standard errors in each study were estimated using the Bayesian feedback method. When a covariate had a significant impact on the model parameters, the original estimates of the model parameters were adjusted by inverse calculation of the covariate functions, which was aimed at deducting the varying degrees of impact of the covariates across different studies.

We then conducted a subgroup analysis of the OS for the three chemotherapy regimens. Specifically, the corrected model parameters were pooled for the three chemotherapy regimens by using a single-arm meta-analysis with a random-effects model to obtain the overall mean and 95% confidence interval (CI). Based on these parameters, the typical value and 95% CI of OS for each chemotherapy regimen were obtained using 10,000 Monte Carlo simulations.

### Objective response rate and safety analysis

A single-arm meta-analysis was performed to summarize the ORR and incidence of grade 3–4 AEs using a random-effects model, from which the typical values and 95% CIs for the three chemotherapy regimens were obtained.

### Software

Model estimation and simulation were performed using NONMEM 7.4 (ICON Development Solutions, United States). The meta-analysis was performed using Stata 14.0 (StataCorp LP, College Station, TX 77845, United States). Diagnostic graphics were drawn using R (version 3.5.1; The R Foundation of Statistical Computing).

## Results

### Characteristics of the included studies

**FIGURE 1:**
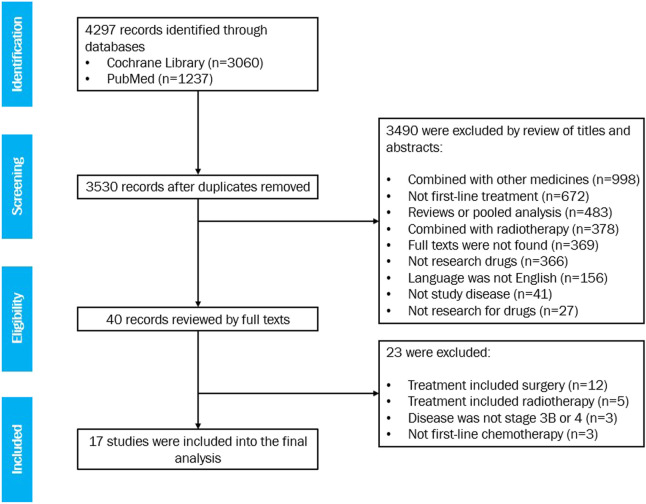
Flow chart demonstrating the literature search and selection.

Seventeen studies met the eligibility criteria, with 20 treatment arms involving 1,368 patients in total (Figure 1). For the list of included studies, see Supplementary Information. Six arms with a sample size of 556 were treated with gemcitabine and vinorelbine, six arms with a sample size of 371 were treated with gemcitabine and docetaxel, and eight arms with a sample size of 441 were treated with gemcitabine alone.

Among the 17 studies, the sample size ranged from 19 to 215 (median 53); the median age of patients ranged from 59.0 to 76.0 years (median 72.5 years); the proportion of male patients was 57.0–90.0% (median 80.7%); the proportion of patients with a PS score of 2 (PS2) was 0.0–100.0% (median 14.0%); the proportion of patients with stage IV disease was 61.9–100.0% (median 79.0%); the proportion of patients with squamous cell carcinoma was 12.0–50.0% (median 34.0%); the proportion of patients with adenocarcinoma was 27.4–58.0% (median 41.1%); and the proportion of patients with large cell carcinoma was 4.0–21.0% (median 9.8%) (Table 1). A summary of the included studies is presented in Supplementary Table S1.

**TABLE 1:** Baseline characteristics of the included studies, median (min-max).

	Overall	Regimens			
		GV	GD	G	*P*
Number of arms	20	6	6	8	-
Total sample size	1358	556	371	441	-
Sample size per arm	53 (19–215)	62 (20–215)	51 (19–144)	47 (28–122)	>0.05
Age (years)	72.5 (59.0–76.0)	61.4 (59.0–65.0)	68.8 (61.4–76.0)	75.0 (72.0–76.0)	<0.05
Male (%)	80.7 (57.0–90.0)	78.3 (59.0–86.0)	83.0 (66.0–90.0)	81.3 (57.0–86.9)	>0.05
PS2 (%)	14.0 (0.0–100.0)	12.0 (2.0–26.0)	11.1 (0.0–19.6)	35.3 (20.5–100.0)	>0.05
Disease stage (%)					
ⅢB	21.0 (0.0–38.1)	15.0 (7.0–33.0)	13.0 (12.0–35.0)	31.5 (4.0–38.1)	>0.05
Ⅳ	79.0 (61.9–100.0)	85.0 (67.0–93.0)	87.0 (65.0–100.0)	68.5 (61.9–96.0)	>0.05
Histological type (%)					
SCC	34.0 (12.0–50.0)	29.7 (21.0–40.0)	35.7 (12.0–38.0)	40.2 (18.0–50.0)	>0.05
ADC	41.1 (27.4–58.0)	53.0 (46.0–55.0)	43.0 (30.4–58.0)	38.3 (27.4–57.0)	<0.05
LCC	9.8 (4.0–21.0)	7.0 (4.0–10.5)	12.0 (7.1–16.0)	8.9 (4.9–15.4)	>0.05

GV, gemcitabine combined with vinorelbine; GD, gemcitabine combined with docetaxel; G, gemcitabine alone; PS2, the proportion of patients with a performance status score of two; SCC, squamous cell carcinoma; ADC, adenocarcinoma; LCC, large cell carcinoma.

All 17 studies showed modified Jadad scores ≥4 (medium to high quality) (Supplementary Tables S2 and S3).

### Model establishment and assessment

The constant hazard function was considered the best for the OS data fitting. We found that age and PS scores significantly influenced mortality risk (Table 2). The equation for the covariate model was as follows:

$$h{\left(t\right)}_{i}=0.098\times e^{\left(\left(AGE_{i}-72.5\right)\times 0.017+\left(PS2_{i}-14\right)\times 0.004\right)}$$ (9)

**TABLE 2:** Parameters of final model.

	Value (RSE%)	95% CI
Parameters		
β_0_	0.098 (6.2)	0.086–0.110
Age on β_0_	0.017 (35.6)	0.005–0.029
PS2 on β_0_	0.004 (35.4)	0.001–0.007
Variability parameters		
η (β_0_), %	14.6 (17.1)	9.7–19.5
ε	0.822 (7.8)	0.696–0.948

RSE is relative standard error; CI is confidential interval; β_0_ is the initial death risk; age on β_0_ is the influence degree of age on β_0_; PS2 on β_0_ is the influence degree of PS2 on β_0_; η is the inter-study variability; ε is the residual error.

In Eq. 9, h(t)_i_ is the death risk at time t of study i; the typical value for the initial death risk for all the studies was 0.098; AGE_i_ is the median age in the *i*th study; the median age for all the studies was 72.5; the correction coefficient of the age to the death risk was 0.017; PS2_i_ is the proportion of patients with PS2 in the *i*th study; the median proportion of patients with PS2 in the overall studies was 14; and the correction coefficient of the proportion of patients with PS2 to the initial death risk was 0.004.

The model showed that for every 10 years of age, the death risk increased by 18.5% (e^10*0.017^ = 1.185), and for every 10% increase in the proportion of patients with PS2, the death risk increased by 4.08% (e^10*0.004^ = 1.0408).

**FIGURE 2:**
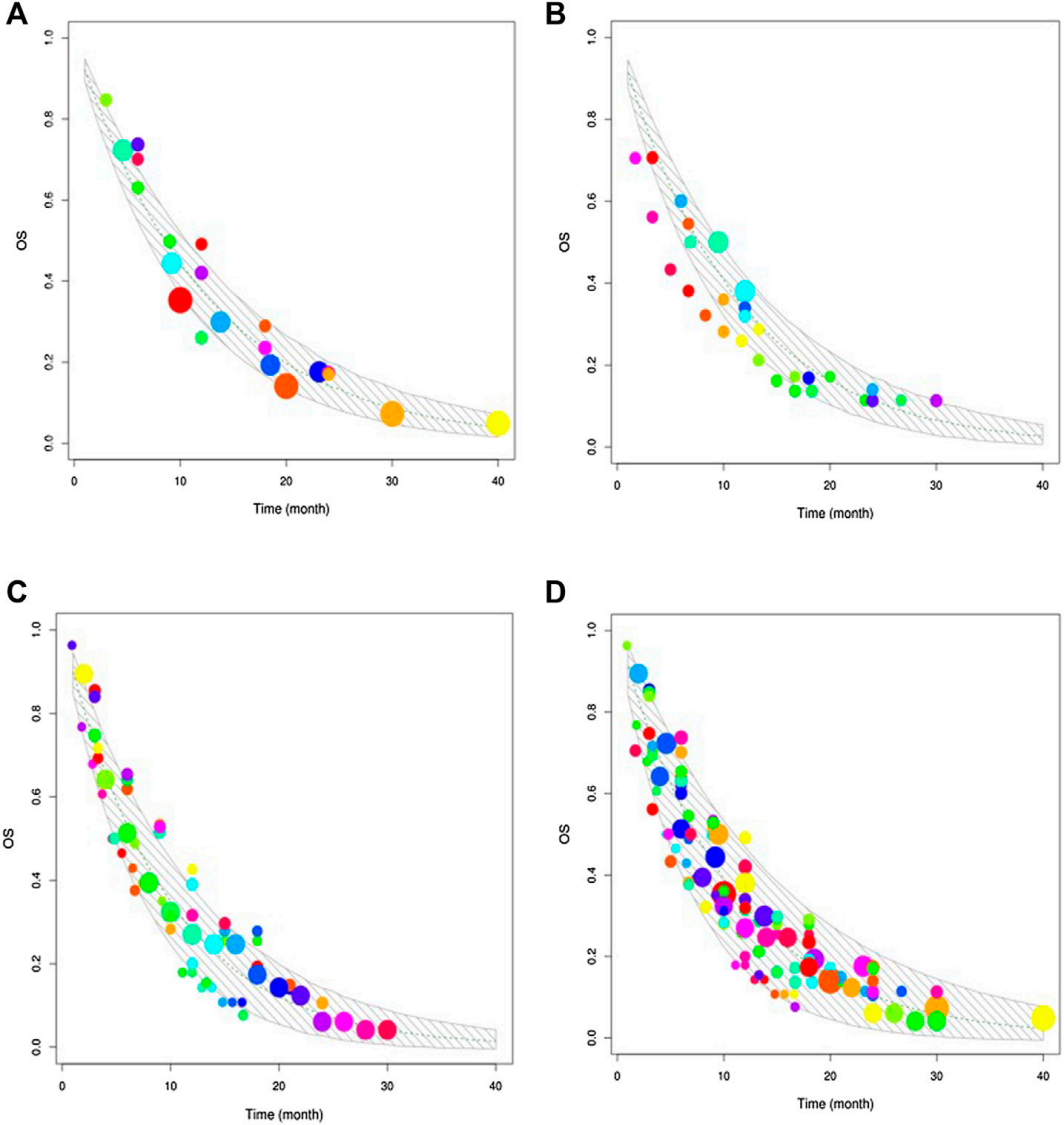
Visual predictive check. The upper and lower solid grey lines stand for the 95% CI of simulated data. The dashed line represents the typical value of simulated data. The points are the observed data, and the point size reflects the corresponding sample size of each study. **(A)** Visual predictive check of gemcitabine combined with vinorelbine. **(B)** Visual predictive check of gemcitabine combined with docetaxel. **(C)** Visual predictive check of gemcitabine alone. **(D)** Visual predictive check of the three regimens.

Table 2 presents the final model parameter estimates. The RSEs of all parameters were less than 40%, indicating that the estimation of the parameters was robust. The diagnostic graphics showed that the model had satisfactory goodness of fit for the data collected from the literature (Supplementary Figures S1 and S2). The Monte Carlo simulation showed that most of the observed data fell within 95% CI of the predicted value, indicating the good predictive capability of the model (Figure 2). Leave-one-out cross-validation showed that the model parameters were stable and slightly affected by the differences in the features of the studies (Supplementary Figure S3).

### Typical overall survival comparison

**FIGURE 3:**
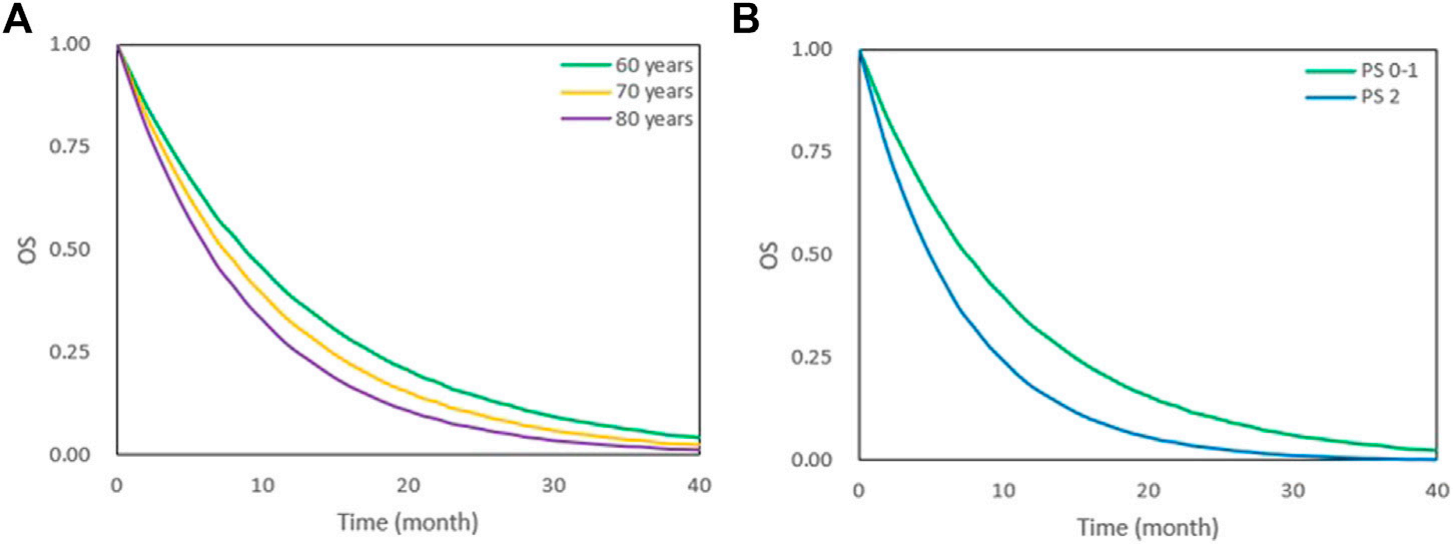
OS under different age and PS scores. **(A)** OS under 60,70 and 80 years old. **(B)** OS under PS 0–1 and PS 2. The lines represent typical efficacies.

During covariate model development, age and PS scores were significant predictors of OS. We simulated the OS of patients classified according to age (60, 70, 80 years) and PS score (PS0–1, PS2) (Figure 3). Younger patients had better OS. The median survival time for patients who were 80 and 60 years old were 6.2 and 8.8 months, respectively. In addition, a lower PS score was associated with better OS. The median survival time of patients with PS0–1 and PS2 were 7.4 and 4.9 months, respectively (Table 3).

**TABLE 3:** MST, 1-year and 2-years survival rate under different age and PS scores, median (min-max).

	MST (month)	1-year survival (%)	2-years survival (%)
Age (years)			
60	8.8 (5.9–12.3)	38.3 (26.4–51.4)	14.8 (6.1–26.0)
70	7.2 (4.9–10.7)	32.1 (20.6–45.1)	10.6 (3.8–20.3)
80	6.2 (4.2–9.0)	25.7 (15.0–38.3)	6.9 (1.5–14.9)
PS			
0-1	7.4 (5.1–10.9)	32.5 (21.4–45.5)	10.7 (3.9–20.5)
2	4.9 (3.4–6.9)	18.1 (9.0–29.5)	3.1 (0–9.1)

MST, median survival time.

**FIGURE 4:**
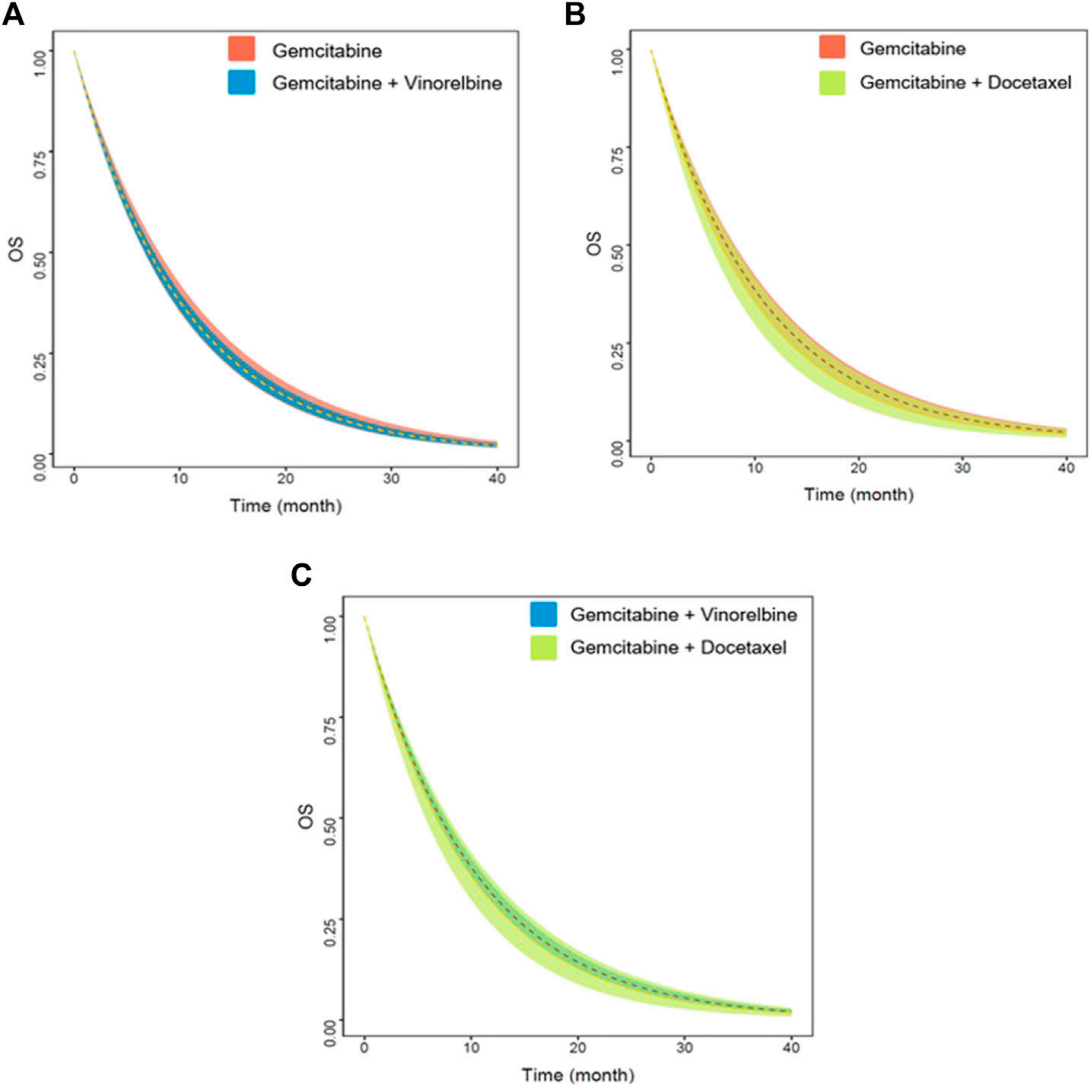
Predicted OS of three non-platinum chemotherapy regimens. **(A)** Gemcitabine vs. gemcitabine combined with vinorelbine. **(B)** Gemcitabine vs. gemcitabine combined with docetaxel. **(C)** Gemcitabine combined with vinorelbine vs. gemcitabine combined with docetaxel. The dashed lines represent the typical efficacies, and the shaded areas are their 95% CIs.

Based on the final model, we also simulated the typical OS and 95% CI of the three first-line non-platinum chemotherapy regimens. When age was adjusted to 72.5 years and the proportion of patients with PS2 to 14%, there were no significant differences among the three regimens (Figure 4). The median survival time for gemcitabine alone was 7.3 (95% CI: 6.7–8.0) months. The median survival time of gemcitabine combined with vinorelbine and gemcitabine combined with docetaxel were 7.1 (95% CI: 6.7–7.5) and 6.7 (95% CI: 5.7–7.8) months, respectively (Table 4).

**TABLE 4:** Model simulation: MST and survival rate of three non-platinum chemotherapy regimens, median (min-max).

Regimens	MST (month)	1-year survival (%)	2-years survival (%)	3-years survival (%)
GV	7.1 (6.7–7.5)	31.2 (28.5–33.2)	9.6 (8.2–11.1)	3.0 (2.2–3.7)
GD	6.7 (5.7–7.8)	28.7 (23.2–34.7)	8.3 (5.3–11.5)	2.3 (1.2–4.1)
G	7.3 (6.7–8.0)	31.5 (28.5–35.5)	10.0 (8.0–12.6)	3.0 (2.2–4.3)

MST, median survival time; GV, gemcitabine combined with vinorelbine; GD, gemcitabine combined with docetaxel; G, gemcitabine alone.

### Objective response rate

Meta-analysis showed that the ORR of gemcitabine alone was 13.8% (95% CI: 10.2–17.2%), which was significantly lower than that of gemcitabine combined with vinorelbine and gemcitabine combined with docetaxel (Table 5). The ORR of gemcitabine combined with vinorelbine was comparable to that of gemcitabine combined with docetaxel, with a value of approximately 28%.

**TABLE 5:** ORR and incidence of grade 3–4 AEs of three non-platinum chemotherapy regimens, median (min-max).

	GV (%)	GD (%)	G (%)
Objective response rate			
ORR	28.4 (17.0–39.8)	28.5 (20.3–36.8)	13.8 (10.3–17.2)
Hematological toxicity			
Anemia	2.1 (0.9–3.3)	5.1 (1.7–8.5)	4.7 (2.3–7.2)
Leukopenia	8.3 (3.7–12.8)	15.0 (8.2–21.9)	11.6 (3.1–20.0)
Neutropenia	20.9 (14.5–27.3)	16.4 (7.4–25.4)	18.7 (7.6–29.7)
Thrombocytopenia	3.0 (1.6–4.5)	4.6 (2.5–6.7)	3.4 (1.2–5.6)
Non-hematological toxicity			
Nausea or Vomiting	2.7 (1.0–4.5)	2.6 (0.8–4.4)	3.6 (0.7–6.5)
Fatigue or Asthenia	6.7 (0.0–14.6)	9.3 (3.3–15.4)	3.3 (0.0–7.0)

AE, adverse event; GV, gemcitabine combined with vinorelbine; GD, gemcitabine combined with docetaxel; G, gemcitabine alone.

### Safety analysis

A summary of grade 3–4 (hematological and non-hematological) AEs associated with the three chemotherapy regimens is provided in Table 5. There were no significant differences in the incidence of anemia, leukopenia, neutropenia, thrombocytopenia, nausea or vomiting, fatigue or asthenia between the three regimens.

## Discussion

The combination of platinum drugs and third-generation chemotherapy drugs is the standard first-line chemotherapy regimen for advanced NSCLC. Platinum drugs are effective, with cisplatin being associated with better OS than carboplatin when combined with third-generation chemotherapy drugs. The most serious AEs associated with cisplatin are nephrotoxicity, gastrointestinal reaction (nausea, vomiting, diarrhoea, or constipation), and mild bone marrow suppression. Carboplatin has no serious nephrotoxicity; however, bone marrow suppression is more pronounced compared to cisplatin. Platinum drugs in combination with third-generation chemotherapy drugs are recommended for younger and relatively fit patients. Non-platinum chemotherapy regimens with milder side-effects are widely used in older patients and in patients with poor physiological functions. Gemcitabine combined with vinorelbine, gemcitabine combined with docetaxel, and gemcitabine alone are the three regimens frequently used as first-line treatment in clinical practice. However, the NCCN guidelines do not specify the use of non-platinum chemotherapy regimens under certain circumstances based on efficacy and toxicity. It is also unclear whether age, sex, PS score, TNM stage, and tumour pathology affect the efficacy of these regimens. Therefore, a comparative assessment of the efficacy and toxicity of the three first-line non-platinum chemotherapy regimens is necessary, and the factors influencing their efficacy need to be explored.

In this study, we found differences in clinical efficacy (OS and ORR) across the three chemotherapy regimens. OS is considered the gold standard for evaluating the efficacy of cancer drugs. However, waiting for an OS as an end-point delays clinical decision-making. ORR indicates the tumour burden after treatment in patients with solid tumours. The responsiveness of a tumour to any type of treatment is reflected in the ORR (Rose, 2004). ORR is an important parameter that demonstrates the efficacy of a treatment, and serves as a primary or secondary endpoint in clinical trials (Aykan and ÖZATLı, 2020). In this study, we found that the ORR of gemcitabine combined with vinorelbine or docetaxel was almost twice that of gemcitabine alone, indicating superior tumour shrinkage when vinorelbine or docetaxel was combined with gemcitabine. In addition, we found that two-drug combinations of gemcitabine did not significantly increase the incidence of grade 3–4 AEs, regardless of haematological or non-haematological toxicity. This may be due to the differences in age and PS scores between patients in two-drug combination group and single-drug group. In the two-drug combination group, the age of patients ranged from 59 to 76 years old and the portion of patients with PS2 ranged from 0 to 26%, while the age of patients ranged from 72 to 76 years old and the portion of patients with PS2 ranged from 0 to 100% in the single-drug group. It showed that younger patients in the two-drug combination group with better physiological function had a better response, and could deal with the toxicity of the two-drug combination better resulting in no significant increase of the incidence of grade 3–4 AEs. In terms of the gold standard OS, the two-drug combination of gemcitabine did not significantly prolong the OS; the median survival time of all three chemotherapy regimens was approximately 7 months. In case of advanced NSCLC, the correlation between ORR and OS is low, and a high ORR does not necessarily benefit OS.

Of third-generation chemotherapy drugs, gemcitabine has been the most extensively researched in clinical trials, and probably the most valuable agent for the treatment of NSCLC. Gemcitabine is suitable for combination therapy because of its unique mechanism of action and nonoverlapping toxicity profile with other third-generation chemotherapy drugs. The main toxicity of gemcitabine is thrombocytopenia. Combination of vinorelbine, or docetaxel with gemcitabine on days 1 and 8 every 3 weeks is a promising administration approach which not only protects against gemcitabine-associated thrombocytopenia but also decreases the incidence of severe neutropenia (Natale, 2001; Langer et al., 2005). The toxicity profile of this regimen was quite favourable, with minimal grade 3–4 AEs aside from granulocytopenia. In third-generation chemotherapy drugs, no other drug is better tolerated than vinorelbine. Tolerance of the drug did not result in a decrease in the elderly subgroup (Piccirillo et al., 2010). Docetaxel, originally developed for the treatment of breast cancer, is highly active in lung cancer (Belani and Eckardt, 2004). Docetaxel has undergone extensive evaluation and is the only third-generation drug approved for first-line and second-line treatment of advanced NSCLC. The combination of gemcitabine with docetaxel has also demonstrated efficacy and safety. A meta-analysis showed comparable survival rates between gemcitabine in combination with docetaxel and platinum-based regimens in first-line treatment of advanced NSCLC, and less grade 3–4 nausea or vomiting, anemia, neutropenia, and febrile neutropenia (Yu et al., 2012). In this study, based on the results of the analysis of efficacy (OS and ORR) and toxicity, the two-drug combination regimens (Gemcitabine combined with vinorelbine, gemcitabine combined with docetaxel) were recommended for patients in good physical condition. These two regimens obtained better ORR, allowing the tumor to shrink, and perhaps creating the opportunity for surgical resection. This result is consistent with a previous study which demonstrated that gemcitabine in combination with a third-generation chemotherapy drug appeared to be more effective and feasible compared with single-drug regimens in the treatment of elderly patients with advanced NSCLC (Russo et al., 2009).

We found that age and PS were the two most important factors affecting OS. Younger patients and those with lower PS scores had higher OS. The median survival time of 60-year-old patients was 2.6 months longer than that of 80-year-old patients, and the median survival time of patients with PS0–1 was 2.5 months longer than that of patients with PS2. A clinical trial exploring the impact of patient selection, based on age, PS score, and comorbidity on the efficacy of the docetaxel and gemcitabine combination showed that patient selection based on these three aspects allowed more appropriate treatment of older patients with NSCLC (Lecaer et al., 2007). In addition, the impact of age and PS scores on OS was associated with immunotherapy for advanced NSCLC (Gil and Um, 2020). A retrospective analysis showed that the median survival time and progression-free survival decreased with an increase in PS scores; patients aged <70 years had a lower rate (7.6%) of immune-related AEs requiring steroids than patients aged ≥70 years (15%). It can be deduced that age and PS scores can act as guidance standards for appropriate treatment selection, as well as predictors of treatment outcomes for advanced NSCLC. In addition, the heterogeneity of age and PS scores should be considered in cross-study comparisons and sample size estimation in the design of clinical trials.

More than half of the patients with advanced NSCLC are elderly. Physiological functions get challenged with aging. Aging is inextricably associated with decreases in organ function, marrow reserve, and drug clearance, especially the case regarding renal and hematopoietic functions. Elderly patients tend to have more severe hematological toxicity than their younger counterparts when receiving chemotherapy. Besides, elderly patients have more comorbidities requiring multiple medications which may interfere with the chemotherapy drugs metabolism (Repetto and Audisio, 2006). All these conditions make the selection of their optimal treatment daunting. PS scores are a major prognostic factor of survival and quality of life but also a guide for the most appropriate treatment of advanced NSCLC. In this study, we found that younger patients and those with lower PS scores had higher OS suggesting early detection and treatment can lead to a higher survival benefit. Younger age and lower PS scores mean relatively better physiological functions and less comorbidities to some extent. This allows for the acceptance of more potent drugs and better management of drug toxicity.

Previous studies have shown that the OS of patients with advanced NSCLC has ethnicity-related differences due to the influence of genotype, lifestyle, and the level of the health care system, and that the OS of East Asians is significantly longer than that of non-East Asians. However, the above conclusions are based on platinum-containing regimens. Due to the limited number of studies included in this analysis, there was no non-East Asian patients included. It was not possible to explore ethnicity-related differences in OS between East Asian and non-East Asian populations.

In recent years, there have been greater survival benefits for patients with advanced NSCLC, given the advent and adoption of targeted therapies and immunotherapies. However, they are limited by gene expression, immune status, or the cost of treatment. Therefore, chemotherapy remains an important component of NSCLC treatment and is often used as a control in clinical trials to assess the benefits of new therapies. Accordingly, it is still of clinical value to compare the efficacy and safety profiles of different chemotherapy regimens. In this study, the distribution characteristics, and predictors of OS for three first-line non-platinum chemotherapy regimens were evaluated. This supplements the quantitative information for medications and can be used as an efficacy scale or reference standard for sample size estimation in clinical trials and for the interpretation of results of single-arm clinical trials in the future.

In this study, literature search was only conducted in PubMed and Cochrane databases, and some literature may have been left out. However, considering the broad representation of literature included in PubMed and Cochrane databases, missed literature have little influence on the conclusions.

## Conclusion

In this study, three first-line non-platinum chemotherapy regimens for advanced NSCLC were quantitatively analyzed for the first time based on OS, and their ORR and toxicity were compared. There were no differences in survival and toxicity; however, younger, and healthier patients who could tolerate the two-drug combination could gain a better ORR. We also found that patient age and PS had a significant impact on patient survival. Older patients with NSCLC and those with higher PS scores had lower OS rates.

## Data Availability

The original contributions presented in the study are included in the article/Supplementary Material, further inquiries can be directed to the corresponding authors.
