# Supplementary material for: Quantitative comparison of the efficacies and safety profiles of three first-line non-platinum chemotherapy regimens for advanced non-small cell lung cancer
Source: Front Pharmacol. 2022 Aug 29;13:806728. doi: 10.3389/fphar.2022.806728 (PMC9465165; doi:10.3389/fphar.2022.806728)
Supplement: Supplementary file 1 [file DataSheet1.docx]

**Supplementary**

**Search Strategies**

**PubMed**

| No. | Query | Results | Date |
| --- | --- | --- | --- |
| #1 | “carcinoma, non-small-cell lung” [mh] OR “non small cell lung carcinoma” [tiab] OR “non small cell lung cancer” [tiab] | 58658 | July-03-2019 |
| #2 | deoxycytidine [mh] OR gemcitabine [tiab] OR Gemzar [tiab] | 28377 | July-03-2019 |
| #3 | vinorelbine [mh] OR vinorelbine [tiab] OR navelbine [tiab] | 3838 | July-03-2019 |
| #4 | docetaxel [mh] OR docetaxel [tiab] OR docetaxol [tiab] OR Taxotere [tiab] | 14311 | July-03-2019 |
| #5 | (“randomized controlled trial” [pt] OR “controlled clinical trial” [pt] OR randomized [tiab] OR placebo [tiab] OR randomly [tiab] OR trial [tiab] OR groups [tiab]) NOT (animals [mh] NOT humans [mh]) | 2210328 | July-03-2019 |
| #6 | English [la] | 23313870 | July-03-2019 |
| #7 | #2 OR #3 OR #4 | 43086 | July-03-2019 |
| #8 | #1 AND #5 AND #6 AND #7 | 2046 | July-03-2019 |
| #9 | Filters: Clinical Trials | 1237 | July-03-2019 |

**Cochrane Library**

| No. | Query | Results | Date |
| --- | --- | --- | --- |
| #1 | MeSH descriptor: [Carcinoma, Non-Small-Cell Lung] explode all trees | 3855 | July-03-2019 |
| #2 | “non small cell lung carcinoma” OR “non small cell lung cancer”: ti, ab, kw | 11824 | July-03-2019 |
| #3 | vinorelbine OR navelbine: ti, ab, kw | 1772 | July-03-2019 |
| #4 | MeSH descriptor: [Deoxycytidine] explode all trees | 3609 | July-03-2019 |
| #5 | gemcitabine OR Gemzar: ti, ab, kw | 5526 | July-03-2019 |
| #6 | docetaxel OR docetaxol OR Taxotere: ti, ab, kw | 6798 | July-03-2019 |
| #7 | #1 OR #2 | 12386 | July-03-2019 |
| #8 | #3 OR #4 OR #5 OR #6 | 15109 | July-03-2019 |
| #9 | #7 AND #8 | 3190 | July-03-2019 |
| #10 | Search limits: Content type: Trials | 3063 | July-03-2019 |

**List of the included studies**

1. Michael, M., et al., Multicenter randomized, open-label phase II trial of sequential erlotinib and gemcitabine compared with gemcitabine monotherapy as first-line therapy in elderly or ECOG PS two patients with advanced NSCLC. Asia Pac J Clin Oncol, 2015. 11(1): p. 4-14.

2. Flotten, O., et al., Vinorelbine and gemcitabine vs vinorelbine and carboplatin as first-line treatment of advanced NSCLC. A phase III randomised controlled trial by the Norwegian Lung Cancer Study Group. Br J Cancer, 2012. 107(3): p. 442-7.

3. Rubio, J.C., et al., A phase II randomized trial of gemcitabine-docetaxel versus gemcitabine-cisplatin in patients with advanced non-small cell lung carcinoma. Cancer Chemother Pharmacol, 2009. 64(2): p. 379-84.

4. Tibaldi, C., et al., First line chemotherapy with planned sequential administration of gemcitabine followed by docetaxel in elderly advanced non-small-cell lung cancer patients: a multicenter phase II study. Br J Cancer, 2008. 98(3): p. 558-63.

5. Gridelli, C., et al., The MILES-2G phase 2 study of single-agent gemcitabine with prolonged constant infusion in advanced non-small cell lung cancer elderly patients. Lung Cancer, 2008. 61(1): p. 67-72.

6. Esteban, E., et al., Pulmonary toxicity in patients treated with gemcitabine plus vinorelbine or docetaxel for advanced non-small cell lung cancer: outcome data on a randomized phase II study. Invest New Drugs, 2008. 26(1): p. 67-74.

7. LeCaer, H., et al., An open multicenter phase II trial of docetaxel-gemcitabine in Charlson score and performance status (PS) selected elderly patients with stage IIIB pleura/IV non-small-cell lung cancer (NSCLC): the GFPC 02-02a study. Crit Rev Oncol Hematol, 2007. 64(1): p. 73-81.

8. Esteban, E., et al., Gemcitabine and vinorelbine (GV) versus cisplatin, gemcitabine and vinorelbine (CGV) as first-line treatment in advanced non small cell lung cancer: results of a prospective randomized phase II study. Invest New Drugs, 2006. 24(3): p. 241-8.

9. Cappuzzo, F., et al., A randomized phase II trial evaluating standard (50 mg/min) versus low (10 mg/min) infusion duration of gemcitabine as first-line treatment in advanced non-small-cell lung cancer patients who are not eligible for platinum-based chemotherapy. Lung Cancer, 2006. 52(3): p. 319-25.

10. Tibaldi, C., et al., Increased dose-intensity of gemcitabine in advanced non small cell lung cancer (NSCLC): a multicenter phase II study in elderly patients from the "polmone toscano group" (POLTO). Lung Cancer, 2005. 48(1): p. 121-7.

11. Quoix, E., et al., First line chemotherapy with gemcitabine in advanced non-small cell lung cancer elderly patients: a randomized phase II study of 3-week versus 4-week schedule. Lung Cancer, 2005. 47(3): p. 405-12.

12. Neubauer, M.A., et al., Results of a phase II trial of gemcitabine in patients with non-small-cell lung cancer and a performance status of 2. Clin Lung Cancer, 2005. 6(4): p. 245-9.

13. Neubauer, M.A., et al., Results of a phase II multicenter trial of weekly docetaxel and gemcitabine as first-line therapy for patients with advanced non-small cell lung cancer. Lung Cancer, 2005. 47(1): p. 121-7.

14. Laack, E., et al., Randomized phase III study of gemcitabine and vinorelbine versus gemcitabine, vinorelbine, and cisplatin in the treatment of advanced non-small-cell lung cancer: from the German and Swiss Lung Cancer Study Group. J Clin Oncol, 2004. 22(12): p. 2348-56.

15. Greco, F.A., et al., Prospective randomized study of four novel chemotherapy regimens in patients with advanced nonsmall cell lung carcinoma: a minnie pearl cancer research network trial. Cancer, 2002. 95(6): p. 1279-85.

16. Georgoulias, V., et al., Comparison of docetaxel/cisplatin to docetaxel/gemcitabine as first-line treatment of advanced non-small cell lung cancer: early results of a randomized trial. Lung Cancer, 2001. 34 Suppl 4: p. S47-51.

17. Bajetta, E., et al., Gemcitabine plus vinorelbine as first-line chemotherapy in advanced nonsmall cell lung carcinoma a phase II trial. Cancer, 2000. 89(4): p. 763-8.

**Table S1.** Summary of the included studies

| **Publication** | **Arm** | **Regimen** | **Sample size** | **Age (year)** | **Male (%)** | **ECOG PS (%)** | | **Stage (%)** | | **Histology (%)** | | |
| --- | --- | --- | --- | --- | --- | --- | --- | --- | --- | --- | --- | --- |
|  |  |  |  |  |  | **0-1** | **2** | **IIIB** | **IV** | **SCC** | **ADC** | **LCC** |
| Fløtten 2012 | 1 | Gemcitabine 1000 mg/m^2^  and Vinorelbine 60 mg/m^2^ | 215 | 65.0 | 59.0 | 74.0 | 26.0 | 15.0 | 85.0 | 26.0 | 55.0 | 4.0 |
| Rubio 2009 | 1 | gemcitabine 1000 mg/m^2^  and Docetaxel 85 mg/m^2^ | 52 | 61.4 | 90.0 | 84.0 | 16.0 | 14.0 | 86.0 | 34.0 | 48.0 | 12.0 |
| Cappuzzo 2006 | 1 | Gemcitabine 1500 mg/m^2^  (Standard 30 min) | 56 | 72.0 | 82.1 | KPS | KPS | 34.0 | 66.0 | 37.5 | 42.9 | 5.4 |
|  | 2 | Gemcitabine 1500 mg/m^2^  (slow infusion 150 min) | 61 | 73.0 | 85.2 | KPS | KPS | 32.7 | 67.3 | 39.3 | 39.3 | 4.9 |
| Esteban 2006 | 1 | Gemcitabine 1250 mg/m^2^  and Vinorelbine 30 mg/m^2^ | 57 | 60.0 | 86.0 | KPS | KPS | 7.0 | 93.0 | 34.0 | 54.0 | NA |
| Laack 2004 | 1 | Gemcitabine 1000 mg/m^2^  and Vinorelbine 25 mg/m^2^ | 143 | 60.8 | 75.5 | KPS | KPS | 12.6 | 87.4 | 29.4 | 49.0 | 10.5 |
| Greco 2002 | 1 | Gemcitabine 1000 mg/m^2^  and Vinorelbine 25 mg/m^2^ | 67 | 62.0 | 69.0 | 88.0 | 12.0 | 27.0 | 73.0 | 21.0 | 46.0 | 9.0 |
| Georgoulias 2001 | 1 | Gemcitabine 1100 mg/m^2^  and Docetaxel 100 mg/m^2^ | 144 | 62.0 | 87.0 | 88.9 | 11.1 | 35.0 | 65.0 | NA | 39.0 | NA |
| Michael 2014 | 1 | Gemcitabine 1000 mg/m^2^ | 28 | 76.0 | 57.0 | 50.0 | 50.0 | 4.0 | 96.0 | 18.0 | 57.0 | 21.0 |
| Gridelli 2008 | 1 | Gemcitabine 1200 mg/m^2^ | 51 | 76.0 | 80.4 | 100.0 | 0.0 | 37.3 | 62.7 | 47.1 | 27.4 | 5.9 |
| Tibaldi 2008 | 1 | Gemcitabine 1200 mg/m^2^  and Docetaxel 37.5 mg/m^2^ | 56 | 76.0 | 82.0 | 80.4 | 19.6 | 0.0 | 100.0 | 35.7 | 30.4 | 7.1 |
| Esteban 2008 | 1 | Gemcitabine 1000 mg/m^2^  and Vinorelbine 25 mg/m^2^ | 20 | 63.0 | 85.0 | KPS | KPS | 15.0 | 85.0 | 40.0 | 55.0 | 5.0 |
|  | 2 | Gemcitabine 1000 mg/m^2^  and Docetaxel 35 mg/m^2^ | 19 | 69.0 | 84.0 | KPS | KPS | 16.0 | 84.0 | 37.0 | 47.0 | 16.0 |
| LeCaer 2007 | 1 | Gemcitabine 900 mg/m^2^  and Docetaxel 30 mg/m^2^ | 50 | 73.7 | 78.0 | 96.0 | 4.0 | 12.0 | 88.0 | 38.0 | 38.0 | NA |
| Tibaldi 2005 | 1 | Gemcitabine 1500 mg/m^2^ | 122 | 75.0 | 86.9 | 79.5 | 20.5 | 30.3 | 69.7 | 44.2 | 32.8 | NA |
| Quoix 2005 | 1 | Gemcitabine 1000 mg/m^2^ | 42 | 75.0 | 85.7 | KPS | KPS | 38.1 | 61.9 | 50.0 | 28.6 | 11.9 |
|  | 2 | Gemcitabine 1125 mg/m^2^ | 39 | 75.0 | 79.5 | KPS | KPS | 28.2 | 71.8 | 41.0 | 38.5 | 15.4 |
| Neubauer Reynolds 2005 | 1 | Gemcitabine 1250 mg/m^2^ | 42 | 73.0 | 59.5 | 0.0 | 100 | 11.9 | 88.1 | 21.4 | 38.1 | NA |
| Neubauer Garfield 2005 | 1 | Gemcitabine 900 mg/m^2^  and Docetaxel 36 mg/m^2^ | 50 | 68.5 | 66.0 | 100.0 | 0.0 | 12.0 | 88.0 | 12.0 | 58.0 | 12.0 |
| Bajetta 2000 | 1 | Gemcitabine 1250 mg/m^2^  and Vinorelbine 25 mg/m^2^ | 54 | 59.0 | 81.0 | 98.0 | 2.0 | 33.0 | 67.0 | 30.0 | 52.0 | NA |

ECOG, Eastern Cooperative Oncology Group; PS, performance status score; SCC, squamous cell carcinoma; ADC, adenocarcinoma; LCC, large cell carcinoma; KPS, Karnofsky performance score; NA, not available.

**Table S2.** The modified Jadad scale

| **No.** | **Question** | **Scoring** |
| --- | --- | --- |
| (1) | Was the study described as randomized? | +1/0 |
| (2) | Was the method of randomization appropriate? | +1/0/-1 |
| (3) | Was the study described as blinding? | +1/+0.5/0 |
| (4) | Was the method of blinding appropriate? | +1/0/-1 |
| (5) | Was there a description of withdrawals and dropouts? | +1/0 |
| (6) | Was there a clear description of the inclusion/exclusion criteria? | +1/0 |
| (7) | Was the method used to assess adverse effects described? | +1/0 |
| (8) | Were the methods of statistical analysis described? | +1/0 |

For question (1), and (5) - (8), award one point for an affirmative response or zero point for a negative response.

For question (2), award one point for an affirmative response; deduct one point if the method of randomization is inappropriate.

For question (3), award one point for double-blind; award 0.5 point for single-blind; award zero point for a negative response.

For question (4), award one point for an affirmative response; deduct one point if the method of blinding is inappropriate.

**Table S3.** Literature quality assessment by modified Jadad scale

| **Study** | **Scoring** | | | | | | | | **Total scores** |
| --- | --- | --- | --- | --- | --- | --- | --- | --- | --- |
|  | **(1)** | **(2)** | **(3)** | **(4)** | **(5)** | **(6)** | **(7)** | **(8)** |  |
| Fløtten 2012 | 1 | 1 | 0 | 0 | 1 | 1 | 1 | 1 | 6 |
| Rubio 2009 | 1 | 1 | 0 | 0 | 1 | 1 | 1 | 1 | 6 |
| Cappuzzo 2006 | 1 | 1 | 0 | 0 | 1 | 1 | 1 | 1 | 6 |
| Esteban 2006 | 1 | 1 | 0 | 0 | 1 | 1 | 1 | 1 | 6 |
| Laack 2004 | 1 | 1 | 0 | 0 | 1 | 1 | 1 | 1 | 6 |
| Greco 2002 | 1 | 1 | 0 | 0 | 1 | 1 | 1 | 1 | 6 |
| Georgoulias 2001 | 1 | 1 | 0 | 0 | 1 | 1 | 1 | 1 | 6 |
| Michael 2014 | 1 | 1 | 0 | 0 | 1 | 1 | 1 | 1 | 6 |
| Gridelli 2008 | 0 | 0 | 0 | 0 | 1 | 1 | 1 | 1 | 4 |
| Tibaldi 2008 | 0 | 0 | 0 | 0 | 1 | 1 | 1 | 1 | 4 |
| Esteban 2008 | 1 | 1 | 0 | 0 | 1 | 1 | 1 | 1 | 6 |
| LeCaer 2007 | 0 | 0 | 0 | 0 | 1 | 1 | 1 | 1 | 4 |
| Tibaldi 2005 | 0 | 0 | 0 | 0 | 1 | 1 | 1 | 1 | 4 |
| Quoix 2005 | 1 | 1 | 0 | 0 | 1 | 1 | 1 | 1 | 6 |
| Neubauer Reynolds 2005 | 0 | 0 | 0 | 0 | 1 | 1 | 1 | 1 | 4 |
| Neubauer Garfield 2005 | 0 | 0 | 0 | 0 | 1 | 1 | 1 | 1 | 4 |
| Bajetta 2000 | 0 | 0 | 0 | 0 | 1 | 1 | 1 | 1 | 4 |


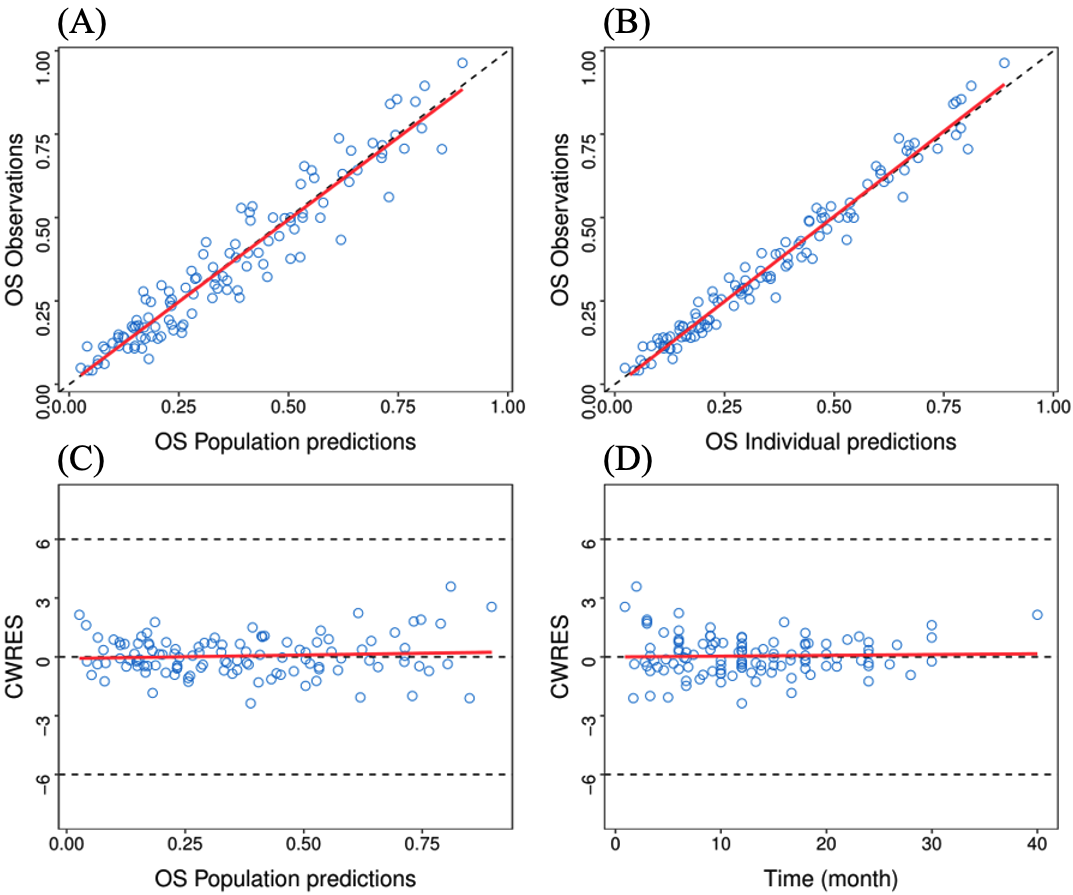


**Figure S1.** Goodness of fit. (A) Population predicted value versus observed value. (B) Individual predicted value versus observed value. (C) Conditional weighted residuals versus population predicted value. (D) Conditional weighted residuals versus time. The solid red lines are the fitted trend line. The dashed black lines in (A) and (B) represent the diagonal lines, whereas in (C) and (D) are the position where conditional weighted residual equals 0 and ±6.


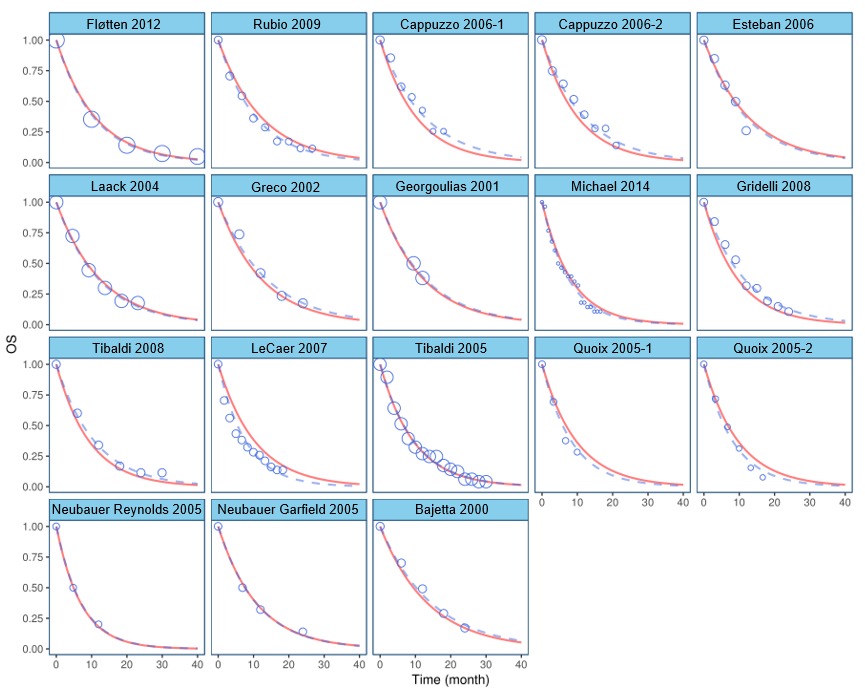


**Figure S2.** Individual time course check. Time course of OS for each individual study. The blue scatter points represent the observed values, and the point size and sample size are positively correlated. The blue dashed lines represent the individual predicted values. The red solid lines represent the population predicted values.


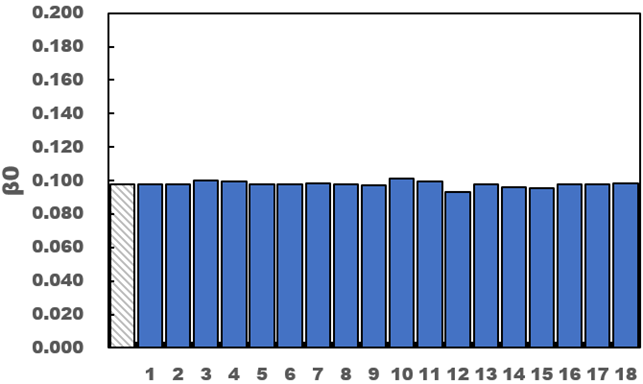


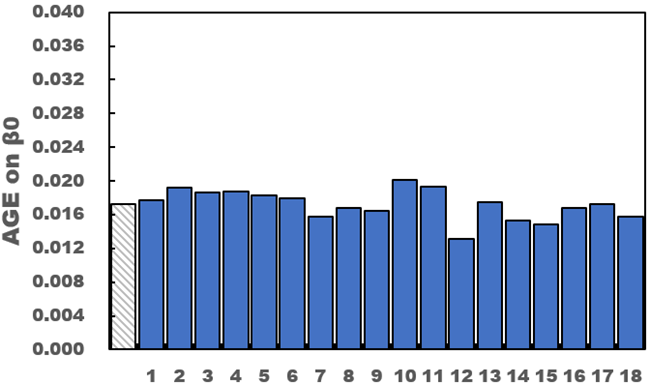


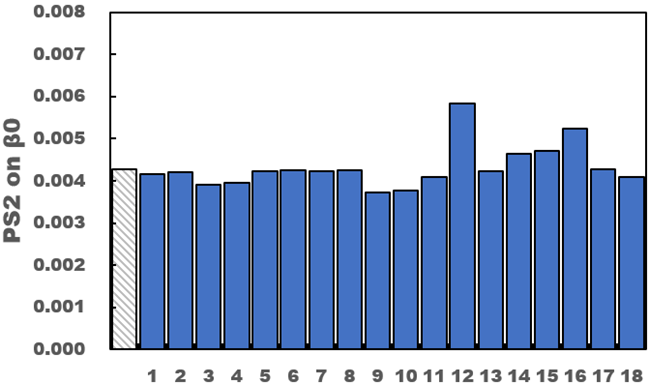


**Figure S3.** Leave-one-out cross validation. Bar charts of the typical parameter values obtained from the final model in leave-one-out method. The abscissa represents the study number dropped from the full data and the ordinate represents the typical value of parameters. The left shadow bar represents parameters obtained from full data. Study 1: Fløtten et al., 2: Rubio et al., 3: Cappuzzo et al., 4: Esteban and Fra et al., 5: Laack et al., 6: Greco et al., 7: Georgoulias et al., 8: Michael et al., 9: Gridelli et al., 10: Tibaldi and Vasile et al., 11: Esteban and Villanueva et al., 12: LeCaer et al., 13: Tibaldi and Ricci et al., 14: Quoix et al., 15: Neubauer and Reynolds et al., 16: Neubauer and Garfield et al., 17: Bajetta et al.

**Table S4.** Single covariate screening results

| **Covariate** | **Covariate model** | **OFV** | **ΔOFV** |
| --- | --- | --- | --- |
| AGE | θ*(AGE-72.5) | -557.297 | -8.316 |
| MALE | θ*(MALE-80.7) | -549.853 | -0.872 |
| PS2 | θ*(PS2-14) | -554.621 | -5.64 |
| STAGE ⅢB | θ*(STAGE ⅢB-21) | -551.036 | 2.055 |
| STAGE Ⅳ | θ*(STAGE Ⅳ-79) | -551.036 | 2.055 |
| SCC | θ*(SCC-34) | -549.028 | 0.047 |
| ADC | θ*(ADC-41.1) | -549.543 | 0.562 |
| LCC | θ*(LCC-9.8) | -555.587 | -6.606 |

OFV, objective functional value; PS2, the proportion of patients with a performance status score of 2; SCC, squamous cell carcinoma; ADC, adenocarcinoma; LCC, large cell carcinoma.

**Table S5.** Covariate screening in a stepwise manner

|  | **Model No.** | **Model description** | **ΔOFV** |
| --- | --- | --- | --- |
| **Forward inclusion** |  |  |  |
|  | 1 | BASE | - |
|  | 2 | 1+AGE | -8.316 |
|  | 3 | 2+PS2 | -3.933 |
|  | 4 | 3+LCC | -7.062 |
| **Backward deletion** |  |  |  |
|  | 5 | 4-LCC | 7.062 |
|  | 6 | 4-PS2 | 2.587 |
|  | 7 | 4-AGE | 8.284 |

OFV, objective functional value; BASE, the base model; PS2, the proportion of patients with a performance status score of 2; LCC, large cell carcinoma. AGE and PS2 were added into the final model based on the changes of OFV and the clinical significance of covariates.
